# Potency of the Combination of Chitosan and Hydroxyapatite on Angiogenesis and Fibroblast Cell Proliferation in Direct Pulp Capping of *Rattus norvegicus*

**DOI:** 10.1055/s-0044-1782212

**Published:** 2024-05-02

**Authors:** Sularsih Sularsih, Wanli Fransiska, Syifa Salsabila, Fitria Rahmitasari, Diana Soesilo, Widyasri Prananingrum

**Affiliations:** 1 Department of Dental Materials, Faculty of Dentistry, Universitas Hang Tuah, Surabaya, Indonesia; 2 Faculty of Dentistry, Universitas Hang Tuah, Surabaya, Indonesia; 3 Department of Conservative Dentistry, Faculty of Dentistry, Universitas Hang Tuah, Surabaya, Indonesia

**Keywords:** chitosan, hydroxyapatite, direct pulp capping, VEGF, blood vessel, fibroblast

## Abstract

**Objectives** The aim of this research was to analyze expression of vascular endothelial growth factor (VEGF), blood vessels, and fibroblast cell proliferation in direct pulp capping treatment of *Rattus norvegicus* using a combination of chitosan and hydroxyapatite paste.

**Materials and Methods** The samples were male *R. norvegicus* strains of Wistar rats, weighing 200 to 250 g and aged between 8 and 16 weeks. The occlusal surface of the molars of *R. norvegicus* was prepared with class I cavity and then perforated with the tip of an explorer. Sixty male *R. Norvegicus* rats were divided into the following: control group (KA) in which the cavity was filled with glass ionomer cement; control group (KB), in which the cavity was filled with Ca(OH)2; PA group, in which the cavity was filled with chitosan (CH); PB group, in which the cavity was filled with hydroxyapatite (HA); and PC group, in which the cavity was filled with chitosan and hydroxyapatite (CH-HA). Each group was divided into 3-, 7-, and 14-day observation groups. The mandibular bone of the molar was cut and histopathological examination was performed to analyze the blood vessels and fibroblast cell proliferation. Immunohistochemistry examination was done to examine the expression of VEGF.

**Statistical Analysis** The data variation was analyzed with One Way Analysis of Variance (ANOVA) test and continued with multiple comparison Least Significant (LSD) test to determine the different pairs of group.

**Results** Analysis of variance (ANOVA) showed a significant increase in the expression of VEGF, blood vessels, and fibroblast cell proliferations ( *p* ≤ 0.05;), especially in the PC group compared to the other four groups. The least significant test (LSD) test showed significant differences between the groups on the expression of VEGF, blood vessels, and fibroblast cell proliferations.

**Conclusion** The combination of chitosan and hydroxyapatite could promote healing of direct pulp capping treatment by increasing the expression of VEGF, blood vessel, and fibroblast cell proliferation.

## Introductions

Caries, trauma, and iatrogenic factors are etiological factors for pulp tissue exposure. When there is pulp tissue exposure, direct pulp caping is considered an efficient conservative treatment option to maintain the vitality of the pulp. It is placed biomaterial directly over an exposed coronal pulp after caries excavation to promote mineralization. The ideal characteristics of a direct pulp capping material are biocompatibility, antibacterial properties, sealing ability, insolubility in tissue fluids, and promotion of the mineralization. 1 2 In previous decades, calcium hydroxide was the gold standard for direct pulp capping treatment. Direct pulp capping materials such as calcium hydroxide and mineral trioxide aggregate (MTA) can promote the migration, proliferation, and differentiation of human dental pulp stem cells. It attempts to seal the pulp by enhancing tertiary dentine deposition. 2 3 However, it has been found that calcium hydroxide can cause superficial coagulation necrosis of the pulp, less adhesive to the dentin, and less successful in long studies. Calcium hydroxide can be ionized into Ca and OH with strong alkali that trigger apical abnormalities. 3 4 Tooth discoloration and high cost of operation are the drawbacks of MTA application. 4 5 Currently, there is no ideal direct pulp capping material. At present, in dentistry various materials are being studied that have the ability to maintain the vitality of the pulp and possess all the ideal characteristics of a direct pulp capping material.

Chitosan (CH) is a natural mucopolysaccharide with properties of biodegradability, biocompatibility, nontoxicity, osteoinductivity, and bacteriostasis, which are suitable for regenerative purposes in dentistry. CH has the potential to promote angiogenesis of human dental pulp stem cells by increasing the expression level of vascular endothelial growth factor (VEGF), fibroblast growth factor (FGF), and angiopoietin-1. 6 7 Therefore, CH is effective and promising for regenerative endodontic. 3

CH is a natural polymer with low mechanical strength that could be blended with hydroxyapatite (HA) and provides a synergistic effect. The physical and mechanical properties of the calcium phosphate compound in HA could improve mechanical properties and osteoconduction of the combination. CH-nanohydroxyapatite scaffold has potential as an effective pulp capping agent to promote odontogenic differential dental pulp stem cells with significant upregulation of VEGF, BMP-2, ALP, and Runx2. 8 9 The pulp is highly vascularized tissue; therefore, healing of injured pulp depends on angiogenesis from angiogenic growth factor, particularly VEGF. Some studies observed that VEGF plays an important role in dentin formation. VEGF can stimulate new blood vessels to regulate migration, proliferation, and odontogenic differention. 3 6 Dental pulp fibroblasts are the most abundant cell type in dental pulp, which plays an important role in dental pulp regeneration. Dental pulp fibroblast secretes VEGF, FGF, and transforming growth factor β1 (TGFβ1), complements proteins when lipoteichoic acid (LTA) stimulates it. These factors are important in promoting dental pulp stem cell (DPSC) migration and growth factor participate in differentiation of DPSC into odontoblastlike cells that generate reparative dentin. 10 The study aims to analyze the expression of VEGF, blood vessels, and fibroblast cell proliferation in direct pulp capping treatment of *Rattus norvegicus* using a combination of CH and HA.

## Materials and Methods

### Preparation of CH, HA, and CH-HA Paste

CH powder with a deacetylation degree of 93% was used in this study. CH powder was synthesized from the tiger prawn ( *Penaeus monodon* ) through deproteinization, depigmentation, and deacetylation (DPA). Three percent CH paste (w/p) was made by dissolving 1.5 g of CH powder in 50 mL of 2% acetic acid (Merck). It was stirred using a magnetic stirrer and then neutralized with NaOH solution (Merck), centrifuged at a speed of 2,000 rpm for 30 minutes, and filtered with filter paper. The gel was added in 3 mL of 0.9% saline and hydroxypropyl methylcellulose (HPMC) solution (Bate Chemical Co., Ltd.) until the consistency of a paste was reached. 11 12

The HA paste was made by dissolving 1.5 g of HA powder (Sigma, Product number: 900204; CAS Number: 1306-06-5) in 50 mL of 2% nitrate acid (Merck). It was then stirred using a magnetic stirrer and added in 3 mL of 0.9% saline and HPMC solution (Bate Chemical Co., Ltd.) until the consistency of a paste was reached. The CH-HA paste was made by mixing CH paste and HA paste in a 50:50 ratio. The mixture was placed in a Petri dish for 24 hours, then added in 3 mL of 0.9% saline and HPMC solution (Bate Chemical Co., Ltd.) until paste consistency was reached. The paste was sterilized using gamma ray radiation with a dose of 1 to 25 kGy. 12

### *In Vivo* Study

This study was experimental research with completely randomized design using samples of molars of male *R. norvegicus* . Rats weighing 200 to 250 g and aged between 8 and 16 weeks were used. The rats were acquired from an experimental animal laboratory of the Faculty of Dentistry, Universitas Hang Tuah, Indonesia. Ethical approval was obtained from the Ethical committee of the Faculty of Dentistry, Universitas Hang Tuah, with certificate number: 022/KEPK-FKGUHT/X/2022. The rats were divided into five groups. There were six samples in each group with 3, 7, and 14 days of observation.

The occlusal surface of the molar of the *R. norvegicus* rat was prepared with class I cavity by using low-speed tapered round diamond burr, and then perforated with the tip of the explorer under anesthesia using ketamine 10% injection (Kepro Pharmacy, the Netherlands) at a dose of 0.1 mL/100 g body weight and xylazine 2% injection (Xyla, Interchemie, the Netherlands) at 0.01 mL/100 g body weight intramuscularly in the upper thigh. 13 14 In the KA control group, the cavity was filled with glass ionomer cement and the cavity was filled with Ca(OH) _2_ in the KB group. In the treatment groups, the PA group, the cavity was filled with CH; in the PB group, the cavity was filled with HA; and in the PC group, the cavity was filled with CH and HA (CH-HA). In the treatment group, after the cavity was filled with CH or HA, it was covered with type IX glass ionomer cement as the restorative material. The *R. norvegicus* rats from each group were sacrificed after 3, 7, and 14 days. The mandibular bone in the interdental region of the mandibular molar was cut and soaked in a fixation solution using 10% formalin buffer. The decalcification process was carried out with ethylenediaminetetraacetic acid (EDTA; J.T. Baker MDL number: MFCD00150037, Fisher Scientific, UK). After that, it was embedded in paraffin blocks. The preparations were made with a thickness of 4 to 5 μm and then continued with hematoxylin and eosin (HE) staining (Sigma Aldrich) in order to observe blood vessels and fibroblast cells in the groups with 3, 7, and 14 days of observation. Immunohistochemical examination was conducted using VEGF monoclonal antibodies (Santa Cruz Biotechnology Inc, [C-1]: sc-726) to observe the expression of VEGF on 3 day of observation. The preparations were observed using a light microscope (Nikom H600L) with 400x magnification in five fields of view.

### Statistical Analysis

The data of the expression of VEGF, blood vessels, and fibroblast cells were statically analyzed using the Shapiro–Wilk test to analyze the normally distributed data. Data homogeneity was analyzed using the Levene test and the differences between the groups were analyzed using one-way analysis of variance (ANOVA) and multiple comparison with the least significant difference (LSD) test ( *p* < 0.05).

## Results

**Fig. 1:**
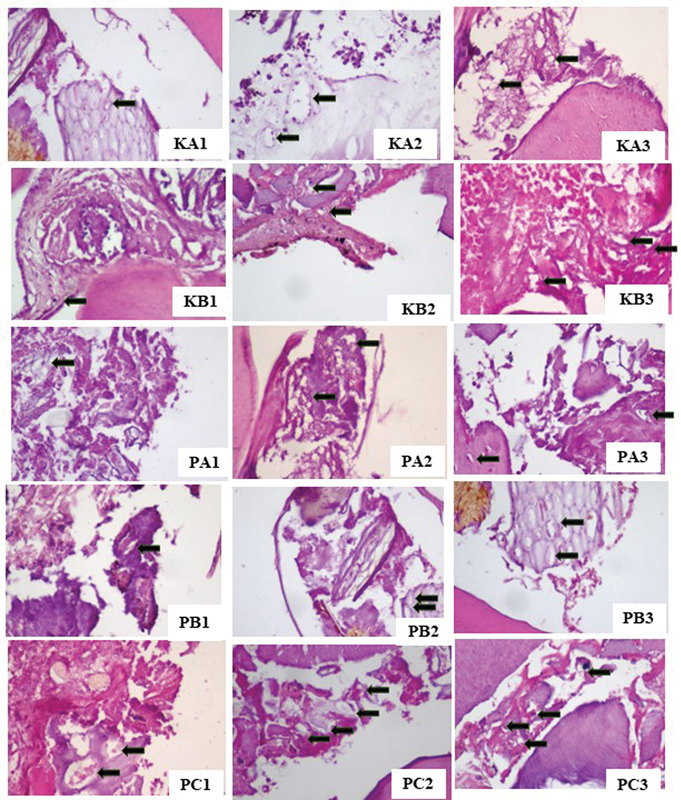
Blood vessels ( *black arrows* ). KA1: glass ionomer cement (GIC) on day 3; KA2: GIC on day 7; KA3: GIC on day 14; KB1: Ca(OH) _2_ on day 3; KB2: Ca(OH) _2_ on day 7; KB3: Ca(OH) _2_ on day 14; PA1: chitosan (CH) on day 3; PA2: CH on day 7; PA3: CH on day 14; PB1: hydroxyapatite (HA) on day 3; PB2: HA on day 7; PB3: HA on day 14; PC1: chitosan and hydroxyapatite (CH-HA) on day 3; PC2: CH-HA on day 7; PC3: CH-HA on day 14, with 400x magnification.

**Fig. 2:**
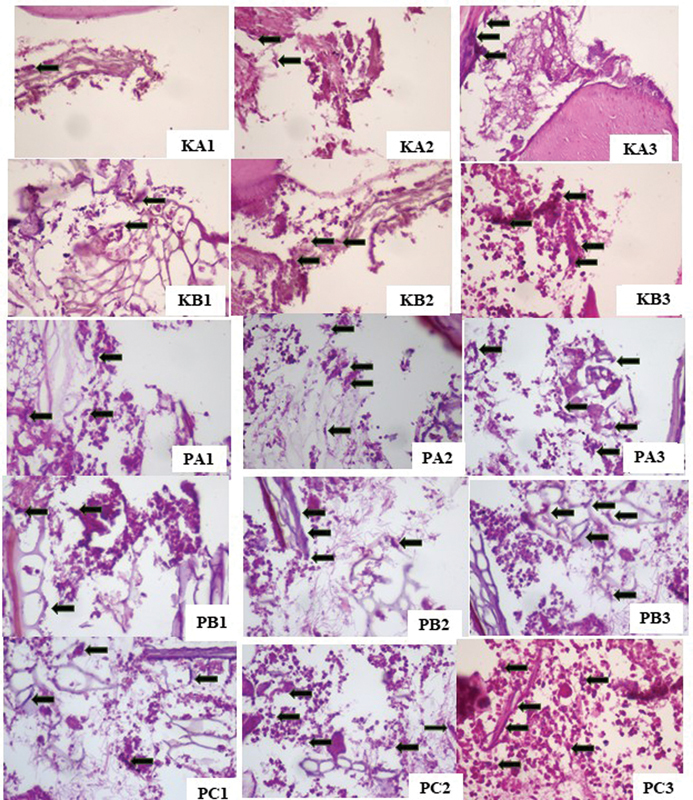
Fibroblast cell proliferation ( *black arrows* ). KA1: glass ionomer cement (GIC) on day 3; KA2: GIC on day 7; KA3: GIC on day 14; KB1: Ca(OH) _2_ on day 3; KB2: Ca(OH) _2_ on day 7; KB3: Ca(OH) _2_ on day 14; PA1: chitosan (CH) on day 3; PA2: CH on day 7; PA3: CH on day 14; PB1: hydroxyapatite (HA) on day 3; PB2: HA on day 7; PB3: HA on day 14; PC1: chitosan and hydroxyapatite (CH-HA) on day 3; PC2: CH-HA on day 7; PC3: CH-HA on day 14, with 400x magnification.

**Fig. 3:**
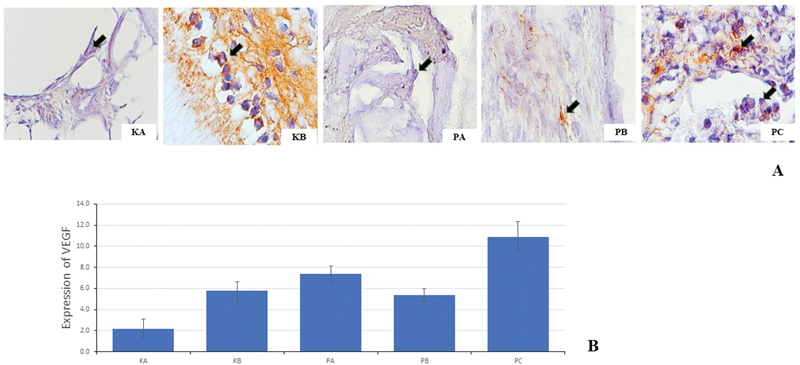
The expression of vascular endothelial growth factor (VEGF) on day 3 ( *black arrows* ). ( **A** ) KA: glass ionomer cement (GIC); KB: Ca(OH) _2_ ; PA: chitosan (CH); PB: hydroxyapatite (HA); PC: chitosan and hydroxyapatite (CH-HA), with 400x magnification. ( **B** ) The increase in VEGF expression was seen the most in the PC group, followed by the PA, KB, PB, and KA groups.

The result of the histopathological examination of the blood vessels and fibroblast cells on days 3, 7, and 14 are shown in Figs. 1 and 2 . The highest blood vessel and fibroblast cell proliferation on healing of direct pulp capping was observed in the PC group with CH-HA, while the lowest was in the KA control group filled with glass ionomer cement ( Tables 1 and 2 ). The PA group using CH and the PB group using HA had a higher number of blood vessels and fibroblast cells than the KA and KB control groups using Ca(OH) _2_ . The result of immunohistochemistry examination of VEGF expression on day 3 is shown in Fig. 3 . The highest expression of VEGF on healing of direct pulp capping was observed in the PC group with CH-HA. It was followed by the PA, KB, PB, and KA groups.

**Table 1:** The average number of blood vessel on days 3,7 and 14

Groups	*N*	Mean of blood vessel ± standard deviation (SD)		
		Day 3	Day 7	Day 14
KA	6	1.33 ± 0.51 ^a^	2.83 ± 0.52 ^b^	3.5 ± 0.53 ^c^
KB	6	2.16 ± 0.75 ^b^	3.5 ± 0.54 ^c^	4.83 ± 0.75 ^d^
PA	6	3.33 ± 0.82 ^c^	4.33 ± 0.81 ^d^	5.98 ± 0.89 ^e^
PB	6	3.0 ± 0.63 ^c^	4.17 ± 0.74 ^d^	5.33 ± 0.51 ^e^
PC	6	4.17 ± 0.75 ^d^	6.33 ± 0.51 ^f^	7.17 ± 0.75 ^g^

Note: a,b,c,d,e,f,g Difference between groups with significance level of 5% ( *p* < 0.05).

**Table 2:** The average number of fibroblast cell proliferation on days 3,7 and 14

Groups	*N*	Mean of fibroblast ± standard deviation (SD)		
		Day 3	Day 7	Day 14
KA	6	1.67 ± 0.52 ^a^	2.67 ± 0.52 ^b^	3.5 ± 0.35 ^c^
KB	6	2.5 ± 0.54 ^b^	3.33 ± 0.52 ^c^	4.5 ± 0.55 ^d^
PA	6	3.33 ± 0.82 ^c^	4.33 ± 0.82 ^d^	5.33 ± 0.52 ^e^
PB	6	3.83 ± 0.75 ^c^	5.0 ± 0.83 ^d^	5.77 ± 0.75 ^e^
PC	6	4.83 ± 0.75 ^d^	6.33 ± 0.52 ^f^	8.0 ± 0.89 ^g^

Note: a,b,c,d,e,f,g Difference between groups with significance level of 5% ( *p* < 0.05).

The data were analyzed using normality test and homogeneity test. The result of analysis was homogenous and had a normal distribution. Blood vessel and fibroblast cell proliferation on healing of direct pulp capping showed significantly differences with the ANOVA test value of *p* = 0.000 ( *p* ≤ 0.05). There were significant differences in blood vessel and fibroblast cell proliferation in the PA, PB, and PC groups compared to the KA and KB groups on days 3, 7, and 14. Between the PA and PB groups, there was not significant difference in blood vessel and fibroblast cell proliferation on days 3, 7, and 14. There was a significant difference in the expression of VEGF in PC group compared to the PA, PB, KA, and KB groups ( *p* = 0.018) on days 3. Between the PA and KB groups, there was no significant difference in the expression of VEGF. The LSD test showed an increase in the expression of VEGF, blood vessel, and fibroblast cell proliferation in the PA, PB, and PC groups.

## Discussion

Direct pulp capping refers to placement of pulp capping material over an exposed coronal pulp to protect the pulp against exposure to promote mineralization for tissue formation. Many dental materials have been studied to preserve pulp vitality. However, currently there is no ideal dental pulp material and it can be challenging to explore materials for pulp treatment. 15 The paramount concern in pulp healing is angiogenesis. It plays a critical role in efficiently transporting various nutrients, chemokines, inflammatory cells, and cytokines. The formation and infiltration of new blood vessel depends on getting signals from angiogenic growth factors, particularly VEGF. 6 16 17 In this study, the expression of VEGF, blood vessel, and fibroblast cell proliferation in the treatment group using CH, HA, and CH-HA was higher than that in the control groups using Ca(OH) _2_ or glass ionomer. There was a significant difference between the groups.

CH is a natural polysaccharide from chitin with properties of nontoxicity, biodegradability, biocompatibility, and osteoinductivity. It also possesses antibacterial properties and is suitable for regenerative purposes in dentistry. CH modulates inflammation and regulates the function of inflammatory cells, macrophages, and fibroblast cells. CH contains active N-acetyl-D-glucosamine dimer, which cross-links with glycosaminoglycan and glycoproteins and activates macrophages to secret growth factors such as VEGF, FGF, TGFβ1, and angiopontin. 3 14 Previous studies reported that loading angiogenic growth factor on biomaterials could enhance pulp tissue healing. VEGF is an angiogenic growth factor that stimulates the formation of new blood vessel. CH is able to stimulate the differentiation of multipotent mesenchymal progenitor cells and growth factor such as FGF and TGFβ1, increasing fibroblast proliferation. 2 7 18 Ca(OH) _2_ been the material of choice for direct pulp capping. However, this material has some deficiencies. Ca(OH) _2_ releases hydroxyl ions that stimulate the expression of nuclear factor kappa β (NF-kβ) and induces proinflammatory protein. Within a few years, the majority of mechanically exposed and capped pulp indicate infection and superficial necrosis after its placement on the exposed pulp. It was probably because of microleakage of the capping materials and tunnel defect in the dentin bridge. It has incomplete setting reaction and ionized into calcium and hydroxide that triggered the risk of internal root resorption and apical lesion. 14 19 When compared to CH and Ca(OH) _2_ , blood vessel and fibroblast proliferations were the lowest in the control group using glass ionomer. There were chronic inflammation and lack of dentin formation caused by persistent acidity of the glass ionomer. 20

Blood vessel and fibroblast cell proliferations in the treatment group using HA were higher than those in the control group using Ca(OH) _2_ or glass ionomer. HA is biocompatible and provides good osseointegration to pulp tissue. It is a porous material that accelerates the angiogenesis process. The calcium phosphate compound in HA could improve the mechanical properties and osteoconduction. It can reduce the production of intercellular fluid and increase the formation of dentin reparative. The calcium phosphate component plays a role in the cell metabolic process by increasing vascularization during pulp healing. It can stimulate the differentiation of stem cell into fibroblast and odontoblastlike cells to promote tissue mineralization and dentin reparative. 21 22 23

CH has desirable characteristics and potential benefits to pulp healing; however, CH has several limitations, such as the fact that it is a crystalline structure arising from solid hydrogen and it has low mechanical strength. 24 It could be blended with HA to provide synergistic effect on the physical and mechanical characteristics. The highest VEGF expression, new blood vessel, and fibroblast cell proliferation on pulp healing were observed in the PC group using CH-HA. The combination of CH-HA a has synergistic effect, resulting a material that has osteoconductive, osteoinductive, and antibacterial properties that have the potential to reduce pulp inflammation and necrosis. 23 25 The calcium phosphate compound of HA could improve mechanical properties and osteoconduction. CH has characteristics of osteoinduction and antibacterial activity. The synergistic effect of its combination promote the pulp healing. The polycationic nature of CH in an acidic solution is provided by the presence of free amino groups within its structure. In addition, the chemical modification of CH is feasible through its amino and hydroxyl groups. The combination promotes mineralized tissue formation, controls infections, prevents microleakage, and maintains the vitality of pulp. 2 7 23 Dental pulp fibroblasts interact with macrophage and modulate their differentiation into M1 (proinflammatory) macrophage to control infection and M2 (anti-inflammatory) to secrete of growth factor such as TGF-β1, FGF-2, VEGF, and complement proteins (C3a and C5a), allowing dental pulp stem cell migration and odontoblastlike cell differentiation. They also secrete angiogenic growth factor for pulp angiogenesis. 10 Thus, the combination of CH and HA can improve the pulp healing process and promote mineralized tissue formation. However, further studies are required to observe the proliferative assays with confocal laser scanning microscopy (CLSM) to approve the degree of dentinal mineralization. Studies to approve the clinical application of CH-HA are also required, exploring combination therapies such as coupling-derived mesenchymal stem cells with growth factors or other regenerative agents to enhance the angiogenic potential in pulp healing. 26 27

## Conclusion

It can be concluded that the combination of CH and HA could accelerate healing of direct pulp capping treatment by increasing the expression of VEGF, blood vessel, and fibroblast cell proliferation.
